# Tuberculous Sacroiliitis Presenting as a Large Gluteal Abscess

**DOI:** 10.7759/cureus.102986

**Published:** 2026-02-04

**Authors:** Jorge-Humberto Ramos-Anguiano, Alejandro J Muñiz-Carvajal, Maria-Patricia Osorio-Navarrete

**Affiliations:** 1 Internal Medicine, Servicios de Salud del Instituto Mexicano del Seguro Social para el Bienestar, Hospital General de Cancún “Dr. Jesús Kumate Rodríguez”, Facultad de Medicina, Universidad Autónoma de Yucatán, Cancun, MEX; 2 Infectious Diseases, Servicios de Salud del Instituto Mexicano del Seguro Social para el Bienestar, Hospital General de Cancún “Dr. Jesús Kumate Rodríguez”, Cancun, MEX; 3 Radiology, Servicios de Salud del Instituto Mexicano del Seguro Social para el Bienestar, Hospital General de Cancún “Dr. Jesús Kumate Rodríguez”, Cancun, MEX

**Keywords:** disseminated tuberculosis, sacro-gluteal sinuses, sacro iliac lesions, skeletal tuberculosis, tuberculosis masquerading

## Abstract

Tuberculous sacroiliitis is a rare form of extrapulmonary tuberculosis. Its nonspecific clinical presentation often leads to delayed diagnosis and treatment, increasing the risk of severe complications such as deformities and functional limitations. We report the case of a 67-year-old male patient who presented with a progressively enlarging gluteal mass and weight loss. Clinical examination revealed a 20 × 20 cm soft, non-tender mass in the right gluteal region, associated with limited joint mobility. Computed tomography (CT) identified osteolytic lesions in the right sacroiliac joint and a collection in the ipsilateral gluteal region, and both lungs showed disseminated micronodules. The collection was drained, and Xpert MTB/RIF (*Mycobacterium tuberculosis*/rifampicin) assay detected *Mycobacterium tuberculosis*. The patient received standard antituberculous therapy for 12 months, resulting in clinical improvement. Tuberculous sacroiliitis requires a high index of suspicion, as delay in treatment may lead to chronic pain and impaired mobility.

## Introduction

Despite advances in diagnosis and treatment, tuberculosis remains an important global health issue, with 10.8 million cases reported in 2023 and an estimated 1.25 million deaths [1]. While most cases of tuberculosis involve the lungs, mycobacteria can disseminate to any tissue. Bone and joint tuberculosis has been reported in 1-3% of all cases [2]. Among these, sacroiliac joint involvement is rarely seen, ranging from 1.7% to 9.7% in older series of skeletal tuberculosis [3,4].

Due to its rarity and nonspecific clinical presentation, diagnosis is often delayed, with series reporting a median time from symptom onset to diagnosis ranging from 10 weeks to eight months [5-8]. This delay may lead to complications such as chronic pain and limitations in mobility due to joint destruction and symptomatic bony ankylosis [2].

In this context, we present a case of tuberculous sacroiliitis presenting as a gluteal abscess, highlighting the importance of maintaining a high index of suspicion for rare presentations of tuberculosis in endemic countries.

## Case presentation

We report the case of a 67-year-old male patient. The patient had a history of chronic alcohol use over the previous 50 years, with increased intake during the last 15 years; he stopped drinking two years before presentation. He reported a fall 15 years earlier that resulted in dislocation of the right knee, for which no medical attention was sought. The knee dislocation was documented during the current presentation, and he had required the use of crutches for ambulation since that time. He reported no chronic diseases, prior surgeries, or known allergies.

The patient presented with a mass in the right gluteal region that had slowly increased in size over 12 months. He also reported unintentional weight loss of 10 kg over the past five years. He denied fever and respiratory symptoms of any kind.

On physical examination, there was a 20 × 20 cm fluctuant, non-tender mass in the right gluteal region, with no overlying skin erythema or local increase in temperature. Baseline laboratory test results are presented in Table 1.

**Table 1:** Initial laboratory test results

Laboratory investigation	Patient value	Normal range	Units
Hemoglobin	11.3	12.5-16	g/dL
White blood cell count	7000	5000-10,500	cells/μL
Hematocrit	32.3	36-52	%
Platelet count	298,000	150,000-400,000	cells/μL
Blood glucose	81	70-100	mg/dL
Serum creatinine	0.6	0.6-1.2	mg/dL
Total bilirrubin	0.47	0.30-1.20	mg/dL
Aspartate aminotransferase	11	15-41	UI/L
Alanine aminotransferase	10	14-54	UI/L
Alkaline phosphatase	63	36-128	UI/L
Erythrocyte sedimentation rate	29	<20	mm/hour
C-reactive protein	22.9	<1.0	mg/dL
HIV antibodies	Non-reactive	Non-reactive	

**Figure 1:**
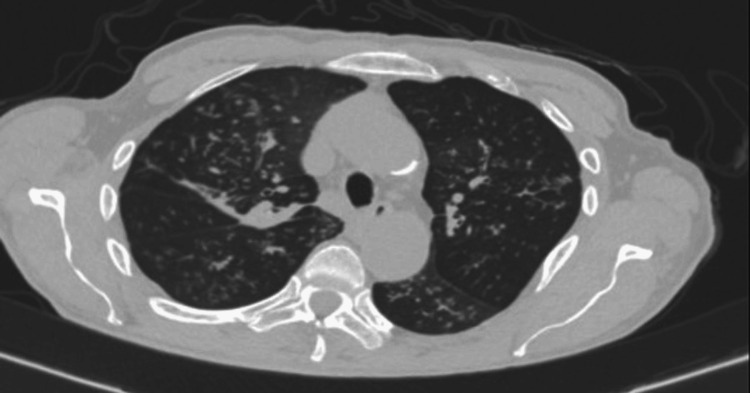
Chest CT showing bilateral micronodules and a tree-in-bud pattern

**Figure 2:**
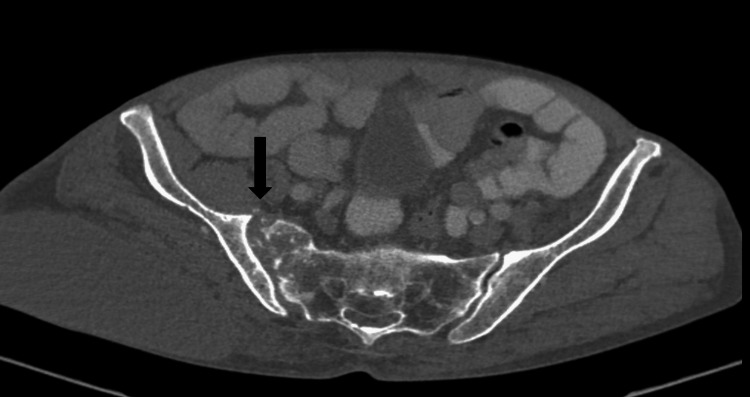
Axial CT of the pelvis, bone window The arrow shows osteolysis of the right sacrum. The left sacroiliac joint shows arthrosis.

**Figure 3:**
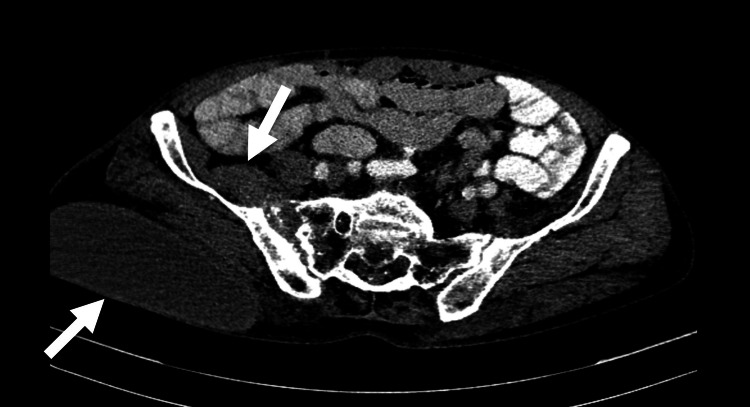
Abdominopelvic axial CT with oral and intravenous contrast CT abdomen demonstrates a hypodense collection arising from the distal portion of the iliopsoas muscle, with extension through a probable fistula to soft tissue of the right gluteal region.

As malignancy was initially suspected, computed tomography (CT) of the chest, abdomen, and pelvis was ordered. Chest CT showed diffuse micronodules and tree-in-bud opacities (Figure 1). Abdominopelvic CT with intravenous and oral contrast demonstrated erosion of the right sacroiliac joint and osteolysis of the right sacrum (Figure 2), with a fluid collection originating from the distal portion of the iliopsoas and extending into the soft tissues of the right gluteal region (Figure 3).

Incision and drainage of the gluteal collection were performed, yielding approximately 1 L of purulent, caseous material. Samples were sent for microbiological analysis; no bacterial growth was observed on culture. Gram and Ziehl-Neelsen stains were negative, and Xpert MTB/RIF (*Mycobacterium tuberculosis*/rifampicin) was positive for *Mycobacterium tuberculosis,* with no rifampin resistance.

Despite the findings on chest CT, the patient did not have any respiratory symptoms. Sputum could not be obtained, even with induction, and bronchoscopy was unavailable at our institution.

Antituberculous therapy was initiated with isoniazid, rifampin, pyrazinamide, and ethambutol for two months, followed by ten months of isoniazid and rifampin, in accordance with local guidelines. Surgical debridement was proposed, but the patient declined.

During monthly evaluations, abscess fluid was drained percutaneously for the first eight months, with volumes ranging from 40 cc in the first month to 9 cc in the eighth month. A sample obtained at eight months was sent for mycobacterial culture, and no growth was reported. The patient completed 12 months of treatment and has remained asymptomatic at two-year follow-up.

## Discussion

The most common symptoms of tuberculous sacroiliitis reported in most series are low back pain and difficulty walking [7-9]. Pain may also involve the lower limb or be described as sciatica [5,9,10]. Our patient did not report either of these symptoms; it is likely that his preexisting limitations masked them. The frequency of constitutional symptoms is variable. Fever is described in 18-70% of patients [5,9], and sweating and weight loss may also occur [7-9], as in our patient.

Gluteal abscesses are very rare in tuberculous sacroiliitis. Prakash et al. mentioned two instances out of 35 patients, including one that required incision and drainage after conservative management failed [8]. Pouchot et al. reported an abscess in the supragluteal region and a gluteal abscess that developed during treatment and resolved after four months of a draining sinus [5]. Our patient did not develop a sinus, possibly because the abscess fluid was drained percutaneously several times during follow-up.

Laboratory results are nonspecific. The erythrocyte sedimentation rate is almost always elevated [5,7,9,10], as in our patient. Mild anemia may be present [5,7], and some patients present with leukopenia [10]. Tuberculin skin testing is frequently positive [5,10] but does not discriminate latent from active infection.

Articular involvement is often unilateral, although bilateral disease can occur [2]. Plain radiographs may disclose loss of articular margins during the early stages, progressing to joint space widening, bony erosions, sclerosis of the joint margins, and periarticular osteopenia [2,5,7]. Computed tomography and magnetic resonance imaging allow better visualization of bone and soft tissue and are superior for evaluating the sacroiliac joint and detecting abscesses [2].

As subcutaneous abscesses are uncommon, microbiological diagnosis usually involves isolation of Mycobacterium tuberculosis from biopsies of the sacroiliac joint or involved tissues. These biopsies may be obtained through closed needle biopsy or open biopsy [2,5]. Mycobacterium tuberculosis can be identified by culture or using polymerase chain reaction techniques [2].

Medical treatment involves combined therapy with isoniazid, rifampin, ethambutol, and pyrazinamide. Reported treatment durations are variable and range from eight to 20 months [7-10]. We chose a 12-month treatment duration in accordance with local guidelines.

Medical treatment alone appears to be sufficient in many cases [7], but patients with suboptimal response to conservative management, advanced disease, or abscess formation may benefit from surgery [6,9,11]. As previously mentioned, the patient declined surgical management.

## Conclusions

Tuberculous sacroiliitis is an unusual cause of chronic low back pain, and diagnosis is usually delayed because of its nonspecific presentation. A high index of suspicion is required, as earlier identification may help avoid complications such as chronic pain and impaired mobility. Rare forms of tuberculosis should be considered in the differential diagnosis of common symptoms in patients who live in or originate from endemic settings.
