# Correction: Tuberculous Sacroiliitis Presenting as a Large Gluteal Abscess

**DOI:** 10.7759/cureus.c435

**Published:** 2026-05-20

**Authors:** Jorge-Humberto Ramos-Anguiano, Alejandro J Muñiz-Carvajal, Maria-Patricia Osorio-Navarrete

**Affiliations:** 1 Internal Medicine, Servicios de Salud del Instituto Mexicano del Seguro Social para el Bienestar, Hospital General de Cancún “Dr. Jesús Kumate Rodríguez”, Cancun, MEX; 2 Infectious Diseases, Servicios de Salud del Instituto Mexicano del Seguro Social para el Bienestar, Hospital General de Cancún “Dr. Jesús Kumate Rodríguez”, Cancun, MEX; 3 Radiology, Servicios de Salud del Instituto Mexicano del Seguro Social para el Bienestar, Hospital General de Cancún “Dr. Jesús Kumate Rodríguez”, Cancun, MEX

This article has been corrected to change all authors’ institutional affiliation to Servicios de Salud del Instituto Mexicano del Seguro Social para el Bienestar, Hospital General de Cancún “Dr. Jesús Kumate Rodríguez”, Cancún MEX.

